# Evaluation of provincial carbon-neutral capacities in the Yellow River basin using DPSIR

**DOI:** 10.1038/s41598-022-23105-z

**Published:** 2022-10-28

**Authors:** Jian Xu, Haiying Wang, Keyu Zhao, Zhi Li

**Affiliations:** 1 Xi’an University of Science and Technology, Xi’an, 710054 China; 2 School of Construction Machinery, Chang’an University, Xi’an, 710064 China

**Keywords:** Environmental social sciences, Environmental economics, Sustainability

## Abstract

The Yellow River basin spans nine provinces and autonomous regions and plays an important role in China's economic and social development and ecological security. However few studies have integrated the concept of carbon neutrality into research to evaluate the carbon-neutral development level. This paper calculates the comprehensive evaluation value of the provincial carbon-neutral capacities comprehensive evaluation index in the Yellow River basin through the driving-force-pressure-state-impact-response (DFPSIR) index system and the global entropy method based on provinces data between 2008 to 2019. The final results indicated that from 2008 to 2019, the carbon-neutral capacities of the provinces in the Yellow River basin were in a state of rapid development and had achieved a grade leap. However, seven provinces had carbon-neutral capacity levels at the Grade III standard in 2019, thereby leaving scope for substantial improvement. Through the above research, we identified the changes in the trend and driving mechanisms of the carbon-neutral capacity of the Yellow River basin and provide a theoretical reference value for a comprehensive realization of carbon neutrality in China in 2060.

## Introduction

Unchecked emissions of greenhouse gases lead to global warming and aggravate problems such as ecological and environmental deterioration and energy crises. Therefore, an increasing number of countries have switched to developing low-carbon economies as an effective way for sustainable development since the advent of the twenty-first century. As a special low-carbon development mode, carbon neutrality promotes the balancing of carbon emissions and carbon absorption through the innovation and reform of technologies and systems, industrial transformation and upgrading, and the promotion and utilization of clean energy. Finally, the coordinated development of society, the economy, the environment, and energy is being emphasized. The terrain of the Yellow River basin is high in the west and low in the east. The western region of the basin is covered with snow throughout the year. Its central area is covered with loess; therefore, the soil and water losses are significant in this region. The eastern region of the basin is comprised mainly of the alluvial plain of the Yellow River. The Yellow River basin spans nine provinces and autonomous regions and plays an important role in China's economic and social development and ecological security. Therefore, the objective and comprehensive evaluation of the carbon-neutral development level of the Yellow River basin is of high significance for the sustainable development of the provinces and cities in the Yellow River basin.

There are few researches on the development level of carbon neutrality, but the establishment and methods of sustainability evaluation system^1–3^, regional low-carbon economy^4,5^, low-carbon transportation^6^, evaluation of ecological level^7^ and analysis of influencing factors of carbon emissions are mature. The evaluation indicators are roughly divided into three categories: basic indicators, core indicators, and different orientation indicators according to the research purpose^8,9^. There are some differences in the selection of indicators by different scholars^10,11^. Jing et al.^12^ collected a set of 21 indicators covering the economic, social and environmental levels, constructed an indicator system, and proposed the comprehensive TOPSIS-ORM method to evaluate the sustainable development performance of shrinking cities in northeast China. Chaofeng et al.^13^ established a low-carbon city construction and evaluation index system in view of the carbon emission problem in urban construction and development. Yuzhao et al.^14^ studied the ecological security index system of the river basin, proposed the improved DPSIR model, and verified the applicability and importance of the improved DPSIR model in the ecological security assessment of the river basin scale. Guo et al.^15^ established the evaluation index system of ULCC from the perspective of driving force and resistance, respectively used the principal component analysis method and cluster analysis method to evaluate and classify the ULCC of Wuhan metropolitan area, and tested the results with fuzzy analytic hierarchy process to find out the problems existing in the city. Duan et al.^16^ constructed the evaluation system of Dalian's low-carbon economic development level based on AHP + entropy weight method. The comprehensive evaluation index of the development level of low-carbon economy in Dalian from 2005 to 2014 was calculated. Cheng et al.^17^ used correlation analysis, fuzzy rough set and entropy weight methods to select and analyze 21 indicators and construct a regional green competitiveness index. The above studies are all studies on evaluation indicators and evaluation methods between low-carbon and related industries or economies in different regions. However, in the evaluation of multi-provincial regions, river basins and heterogeneous regions, the characteristic indicators increase, and further research on the index system and evaluation methods is also needed.

In terms of carbon neutrality capacity evaluation and its influencing factors, Niu et al.^18^ constructed an evaluation index system including 20 indicators at six levels. An improved TOPSIS method is proposed for analysis and calculation, and the results show that the use of renewable energy, the maintenance of ecological environment quality and low carbon technology are the important factors affecting the carbon neutrality ability of China. Liu et al.^19^ used China's provincial panel data from 2004 to 2016 and the Dubin spatial model based on the Stippart model to empirically analyze the impact of the three subsystems of ecological civilization on carbon emission intensity. Cao et al.^20^ proposed an evaluation model based on the improved matting element extension cloud model to evaluate China's ecological and environmental performance in 2019, taking Jiangsu Province as an example. Wei et al.^21^ adopted the PSR method to identify the influencing factors of China's carbon emissions. Jiang et al.^22^ respectively studied the impact of structural changes in internal and external inputs on global carbon emissions, and used structural decomposition analysis to decompose changes in global carbon emissions into six factors: carbon emission intensity, domestic input structure, international input structure, consumption pattern, consumption and population. Wei et. al.^24^ took Xiamen City as an example to establish a calculation method for the carbon footprint accounting of urban buildings. Ghaffar et. al.^25^ establishes baseline information on urban energy use and CO_2_ emissions. Built-up areas verify the increment of urban CO_2_ sources.

In summary, the current research results show that the DPSIR model can effectively represent the concept and structure of the composite system, and can be well applied to the evaluation of low-carbon or carbon neutral development levels in river basins or multi-province regions. However, there are the following problems in the research: (1) most of the research objects of low-carbon development are urban areas or single industries, and there are few studies on the evaluation of low-carbon development level in river basins, multi-provincial or multi-heterogeneous regions. (2) At present, most of the research focuses on the field of low-carbon economy. (3) At present, most of the evaluation indicators of low-carbon development level are aimed at both carbon emission and carbon absorption, and no evaluation indicators have been established for the process of carbon transmission.

In view of the shortcomings in the above studies, this paper builds the DPSIR analysis model of carbon neutrality capacity evaluation on the basis of the existing studies, and integrates the ecological evaluation index according to the actual situation of carbon emission, carbon absorption, economy, population, technology, resources and environment in the Yellow River Basin. Improve the driving mechanism analysis, pressure measurement, state assessment, impact analysis, response evaluation and other index systems of the carbon neutrality evaluation system in the Yellow River Basin. Combined with statistical data, the analytic hierarchy process and entropy weight method are used to evaluate the carbon neutrality capacity of the nine provinces in the Yellow River Basin. To verify the applicability and importance of DPSIR model in the evaluation of carbon neutral development level in the Yellow River Basin, and to provide theoretical reference for promoting the sustainable development of the Yellow River Basin.

## Construction of a provincial carbon-neutral capacity evaluation index system for the Yellow River basin

### DPSIR model

**Figure 1:**
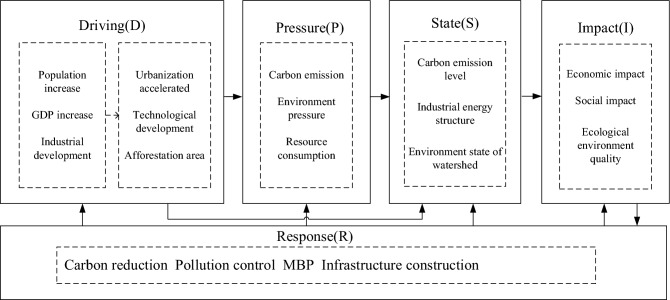
DPSIR model for carbon-neutral capacity evaluation in the Yellow River basin.

The driving-pressure-state-impact-response (DPSIR) model has evolved from the pressure-state-response (PSR) model proposed by the Organization for Economic Cooperation and Development and the driving-pressure-response (DPR) model proposed by the United Nations Commission on Sustainable Development. It is comprehensive, systematic, holistic, and flexible. The model can reveal the causal relationship between the environment and economy. The DPSIR model divides the system into five factors: driving, pressure, status, impact, and response. Figure 1 depicts the DPSIR concept model for the Yellow River basin and reflects the interaction process of carbon-neutral influencing factors. Driving (D) represents the driving factors of carbon-neutral capacity caused by provincial economic development in the Yellow River Basin, and pressure (P) refers to the pressure of the economic development activities on the carbon-neutral capacity of the basin. State (S) is the state level of the provincial carbon-neutral capacity under pressure. Influence (I) is the feedback result and impact of various state levels on the provincial carbon-neutral capacity in the Yellow River Basin. Response (R) refers to various positive measures and countermeasures adopted to enhance the carbon-neutral capacity of provinces in the basin.

### Construction of the carbon-neutral-capacity evaluation index system

TO objectively and scientifically evaluate the carbon-neutral capacity of the Yellow River basin, this study follows the principles of the DPSIR model: science, system, compatibility, regional, hierarchy, operability, and regional economic and social development. The evaluation index system includes three levels: the target layer, subsystem layer, and index layer. The determined index layer comprises 37 specific indicators. It determines its attributes as positive (+) or negative indices (−) for the index characteristics. The specific carbon-neutral-capacity evaluation index system is listed in Table 1.

**Table 1:** Carbon-neutral-capacity evaluation index system for the Yellow River basin.

Target layer	Subsystem layer	Index layer	Variable	Description and description	Properties	Notes
Evaluation of provincial carbon neutral capacity in the Yellow River Basin	Driving (D)	GDP per capita	d_1_	Measure people's living standard (Yuan)	+	^5,9,18,23^
		GDP per capita growth rate	d_2_	Measure the growth rate of regional economic growth (%)	+	^18^
		Provincial population growth rate	d_3_	Measure the regional population growth rate (%)	−	^9,18,23^
		Provincial permanent population	d_4_	Characterizing the provincial population distribution (10,000 people)	−	^18^
		Urbanization level	d_5_	The process and degree of population aggregation toward the city (%)	+	^2,5,9,18,23^
		Industrial output value growth rate	d_6_	Industrial production growth degree (%)	−	^2,18,21^
		Approved patents ratio	d_7_	Regional scientific and technological innovation capacity	+	^2,5^
		Afforestation areas	d_8_	Measure the ecological improvement capacity of the area (ha/person)	+	^2,9^
	Pressure (P)	Energy consumption per capita	p_1_	Capacity to consume energy per person (tons of standard coal/person)	−	^2,18,23^
		Carbon emissions per capita	p_2_	Total carbon emissions per person (ton/person)	−	^20,21,23^
		Cars per capita	p_3_	Measure traffic carbon pollution (vehicle/person)	−	^2,18^
		Average annual heating days	p_4_	Impact of heating (days)	−	^21^
		living area per capita	p_5_	Impact of construction industry (m ^2^/person)	+	^2,18,21,22^
	Status (S)	proportion of renewable energy power	s_1_	Measure the regional dependence on traditional energy generation (%)	+	^2,18^
		Carbon absorption to carbon emission ratio	s_2_	Total regional carbon absorption/total carbon emissions (%)	+	^21,22^
		GDP proportion of the secondary industry	s_3_	GDP of processing and manufacturing industry/GDP of region (%)	−	^2,18,20,22^
		Proportion of national hygiene cities	s_4_	The province was selected as National Health City/all cities in the province (%)	+	^22^
		GDP proportion of the tertiary industry	s_5_	Service sector GDP gross value/regional GDP gross value (%)	+	^2,18,20,22^
		Cultivated land area per capita	s_6_	Per capita cultivated land area (mu/person)	+	^18^
		Regional air qualified rate	s_7_	Number of days the air quality meets the standard/365	+	^18,20,22^
		Annual average flow	s_8_	liquid volume flowing through a section (m^3^/s)	+	^22^
		Water qualified rate	s_9_	Number of days of Yellow River/(%)	+	^22^
	Impact (I)	Annual average temperature change rate	i_1_	Measure the stability of the regional temperature (℃)	−	^21^
		Carbon dioxide concentration	i_2_	Regional air CO_2_ contains (%)	−	^18,20^
		Air pollution index	i_3_	Measures the quality of regional air quality	−	^18,20,22^
		Proportion of soil erosion	i_4_	Provincial soil and soil loss area/provincial total land area (%)	−	^22^
		Comprehensive pollution index of water quality	i_5_	The method of evaluating the water environmental quality is divided into six levels	−	^18,22^
		Waste water discharge volume	i_6_	Total annual wastewater discharge (ton/year)	−	^2,18^
	Response (R)	Proportion of photovoltaic power	r_1_	Solar power generation/total power generation: (%)	+	^2^
		Percentage of the forestry and grass coverage	r_2_	Measure the ability of the natural environment to absorb carbon absorption (%)	+	^2,18,20,22^
		Public transport travel ratio	r_3_	Environmental impact of carbon emissions from traffic (%)	+	^2,20^
		Proportion of wind power	r_4_	Wind power generation/total power generation: (%)	+	^2^
		Comprehensive utilization percentage of industrial solid waste	r_5_	Comprehensive utilization of industrial solid waste accounts for industrial solid waste production (%)	+	^2,18,20,22^
		Centralized treatment rate of urban sewage	r_6_	Percentage of sewage treated by urban centralized sewage treatment plant and urban sewage discharge (%)	+	^20,22^
		Low-carbon economic development plan	r_7_	Government response to carbon neutrality	+	^18,20^
		Urban road areas per capita	r_8_	Reacting the congestion of urban traffic (m^2^/person)	+	^20^
		Hydropower station capacity	r_9_	Measure the dependence on new energy generation (GW)	+	^22^

## Evaluation of provincial carbon-neutral capacity level in the Yellow River basin

### Data source and collection methodology

**Figure 2:**
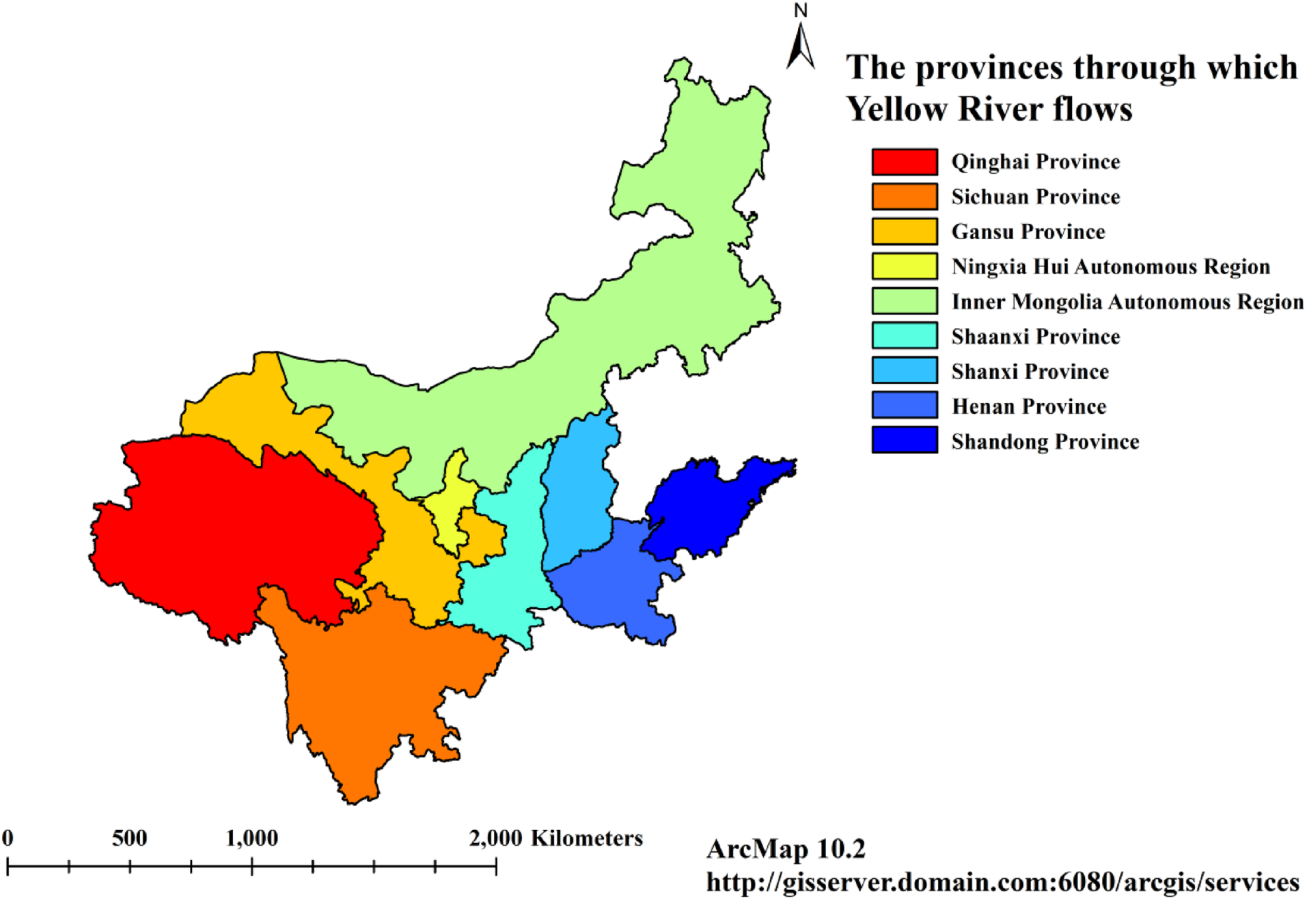
The research areas of the Yellow River Basin.

The research objects of this paper are nine provinces in the Yellow River basin, mainly including Qinghai, Sichuan, Gansu, Ningxia, Inner Mongolia, Shaanxi, Henan, Shanxi,and Shandong. As shown in Fig. 2.

The data on economic and social development were mainly sourced from China Statistical Yearbooks from 2008 to 2019 and included data for nine Yellow River basin provinces. The data on the use of resources were sourced mainly from the China Energy Statistical Yearbooks from 2008 to 2019. The data on environmental quality and environmental governance were sourced mainly from the 2008–2019 China Environmental Statistics Yearbooks, China Bulletin on the Status of the Marine Environment and the Department of Ecology and Environment of the nine Yellow River Basin provinces. The indicator data on per capita carbon emissions, annual average temperature change rate, GDP proportion of secondary industry, tertiary industry GDP proportion, and provincial health cities were calculated using their respective formulas from the index description and explanation given in Table 1.

### Evaluation-index weight determination based on the global entropy value method

After the evaluation index system was established, an appropriate evaluation method was selected to evaluate the development level of the system. Commonly used comprehensive evaluation methods include hierarchical analysis, fuzzy comprehensive evaluation, main component analysis, factor analysis, and entropy methods. The entropy method is an objective empowerment method that calculates the degree of numerical dispersion among the indicators. However, the traditional entropy method can handle only cross-sectional data. It cannot handle the panel data of a multi-index system spanning years. Therefore, this study used the global entropy method to determine the weight of the evaluation index system for the provincial carbon-neutral capacity of the Yellow River basin.

1. Standardized processing of index data Because the original data of the N index may have different units and dimensions, these data must be standardized to obtain standardized data ${\mathrm{z}}_{\upalpha {\mathrm{ij}}}$:
  1 $${\mathrm{z}}_{\upalpha {\mathrm{ij}}} = \left\{\begin{array}{c}\frac{ {\mathrm{x}}_{\upalpha {\mathrm{ij}}}-{\mathrm{x}}_{\mathrm{min}}}{{\mathrm{x}}_{\mathrm{max}}-{\mathrm{x}}_{\mathrm{min}}} \;\; \text{forward indicators}\\ \frac{ {\mathrm{x}}_{\mathrm{max}}-{\mathrm{x}}_{\upalpha{\mathrm{ij}}}}{{\mathrm{x}}_{\mathrm{max}}-{\mathrm{x}}_{\mathrm{min}}} \;\; \text{Negative indicators}\end{array}\right.$$
  where ${{\mathrm{x}}_{\mathrm{min}}\mathrm{ and x}}_{\mathrm{max}}$ represent the minimum and maximum of an index in all years and in all provinces, respectively; ${{\mathrm{x}}_{\upalpha{\mathrm{ij}}}\mathrm{ and z}}_{\upalpha{\mathrm{ij}}}$, respectively, represent the values of item α of the jth province before and after standardization in year i.
2. Normalization of index standardization data:
  2 $${\mathrm{y}}_{\upalpha {\mathrm{ij}}}=\frac{{\mathrm{z}}_{\upalpha{\mathrm{ ij}}}}{\sum_{\mathrm{i}=1}^{\mathrm{m}}\sum_{\mathrm{j}=1}^{\mathrm{n}}{\mathrm{z}}_{\upalpha{\mathrm{ij}}}}$$
3. Calculate the information entropy of each index ${\mathrm{E}}_{{\upalpha }}$:
  3 $${\mathrm{E}}_{{\upalpha }}=-\mathrm{k}\sum \limits _{\mathrm{i}=1}^{\mathrm{m}}\sum \limits_ {\mathrm{j}=1}^{\mathrm{n}}{\mathrm{y}}_{\upalpha{\mathrm{ij}}}{\mathrm{lny}}_{\upalpha{\mathrm{ ij}}}$$
  where $\mathrm{k}=1/\mathrm{ln}(\mathrm{m}\times \mathrm{n})$. When ${\mathrm{y}}_{\upalpha{\mathrm{ ij}}}=0$, set ${\mathrm{y}}_{\upalpha{\mathrm{ ij}}}{\mathrm{lny}}_{\upalpha{\mathrm{ ij}}}$ = 0.
4. Calculate the redundancy of each index ${\mathrm{D}}_{{\upalpha }}$:
  4 $${\mathrm{D}}_{{\upalpha }}=1-{\mathrm{E}}_{{\upalpha }}$$
5. Calculate the weights of each index ${\mathrm{w}}_{{\upalpha }}$:
  5 $${\mathrm{w}}_{{\upalpha }}=\frac{{\mathrm{D}}_{{\upalpha }}}{\sum_{{\upalpha }=1}^{\mathrm{N}}{\mathrm{D}}_{{\upalpha }}}$$

### DPSIR model subsystem level evaluation

The DPSIR model subsystem layer evaluation formula is as follows:

6 $${\mathrm{S}}_{\mathrm{dpsir}}=\sum\limits_{{\upalpha }=1}^{\mathrm{N}}{\mathrm{w}}_{{\upalpha }}\times {\mathrm{y}}_{\upalpha {\mathrm{ij}}}$$

where ${\mathrm{S}}_{\mathrm{dpsir}}$ is the subsystem layer evaluation value of the DPSIR model. The five subsystem evaluation values are as follows: driving subsystem evaluation value (S_d_), pressure subsystem evaluation value (S_p_), state subsystem evaluation value (S_s_), impact subsystem evaluation value (S_i_), and response subsystem evaluation value (S_r_).

### Comprehensive evaluation index and capacity level of provincial carbon-neutral capacity of the Yellow River basin

The weight (W_dpsir_) of the DPSIR subsystem layer was determined using the expert evaluation method. The five subsystem weights were as follows: driving (w_d_), pressure (W_p_), state (W_s_), impact (W_i._) and response (W_r_) weights.

The carbon-neutral capacities comprehensive evaluation Index (CCCEI) was obtained as

7 $$\mathrm{CCCEI}={\mathrm{S}}_{\mathrm{d}}\times {\mathrm{W}}_{\mathrm{d}}+{\mathrm{S}}_{\mathrm{p }}\times {\mathrm{W}}_{\mathrm{p}}+{\mathrm{S}}_{\mathrm{s}}\times {\mathrm{W}}_{\mathrm{s}}+{\mathrm{S}}_{\mathrm{i}}\times {\mathrm{W}}_{\mathrm{i}}+{\mathrm{S}}_{\mathrm{r}}\times {\mathrm{W}}_{\mathrm{r}}$$

Considering the score of the comprehensive evaluation index of the provincial carbon-neutral capacity of the Yellow River basin and referring to the relevant comprehensive index classification method at home and abroad and the actual situation of this study, the grading standards for establishing provincial carbon-neutral capacities in the Yellow River basin are listed in Table 2.

**Table 2:** Classification of carbon-neutral capacities in the Yellow River basin.

CCCEI	Grade	Carbon-neutral capacity level
≤ 0.4	I	Poor
(0.4, 0.8]	II	Fair
(0.8, 1.2]	III	Average
(1.2, 1.6]	IV	Good
> 1.6	V	Excellent

## Empirical analysis

### Analysis of factors affecting provincial carbon-neutral capacity development levels in the Yellow River basin

The DPSIR subsystem was thoroughly analyzed by comparing the evaluation values of the carbon-neutral capacity DPSIR subsystem of the nine provinces in the Yellow River basin from 2008 to 2019 and combining the characteristics of resources and environment and the specific characteristics of the basin.

1. Driving subsystem
  The evaluation values of the carbon-neutral capacity driving subsystems in nine provinces in the Yellow River basin generally exhibited an upward trend, as depicted in Fig. 3. Among the provinces, Inner Mongolia has an extensive and large forest coverage area; therefore, its carbon-neutral driving subsystem evaluation value is at a relatively high level. Sichuan, Shaanxi, and Shandong have the largest GDP per capita growth rate, and relative scientific research patent technological achievements have gradually increased. Economic and technological developments provide sufficient support for carbon neutrality. The evaluation value of the carbon-neutral driving subsystem increased steadily, with a small annual growth in other provinces. The growth rate of various carbon-neutral driving indices slowed. Overall, the carbon-neutral capacity driving subsystem in nine provinces in the Yellow River basin has been maintained at a relatively high level and has achieved good driving results.

**Figure 3:**
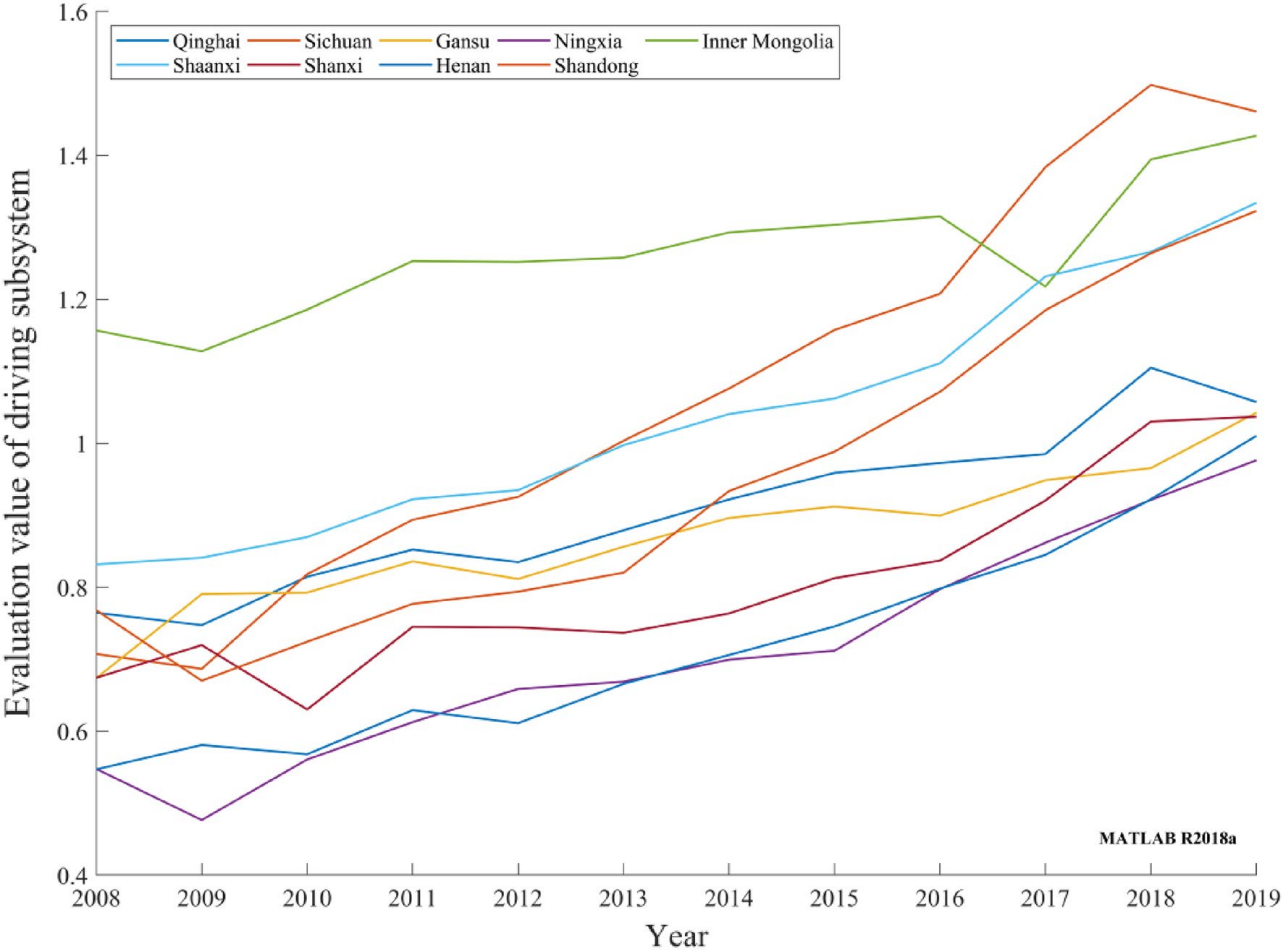
Evaluation values of provincial carbon-neutral driving subsystems in the Yellow River basin during 2008–2019.
2. Pressure subsystem
  Although the evaluation values of the carbon-neutral pressure subsystems in the nine provinces of the Yellow River basin decreased slightly in some years during 2008–2019, the overall trend exhibited a small increase, as depicted in Fig. 4. Carbon emissions per capita increased slightly. However, with economic development and technological progress, the energy consumed per unit economic growth was significantly reduced. Overall, the pressure subsystem evaluation values exhibited an increasing trend. Because the Yellow River basin provinces are located in the north, the heating requirements and people's living conditions put increased pressure on the resources and environment. This leads to the instability of the pressure subsystem evaluation values and inhibits the growth of the pressure subsystem evaluation values. Although the provinces have actively strengthened the idea of a low-carbon economy in recent years and gradually increased their investment in realizing the carbon-neutral development goal, the pressure problems facing carbon neutrality are difficult to improve significantly in a short timeframe.

**Figure 4:**
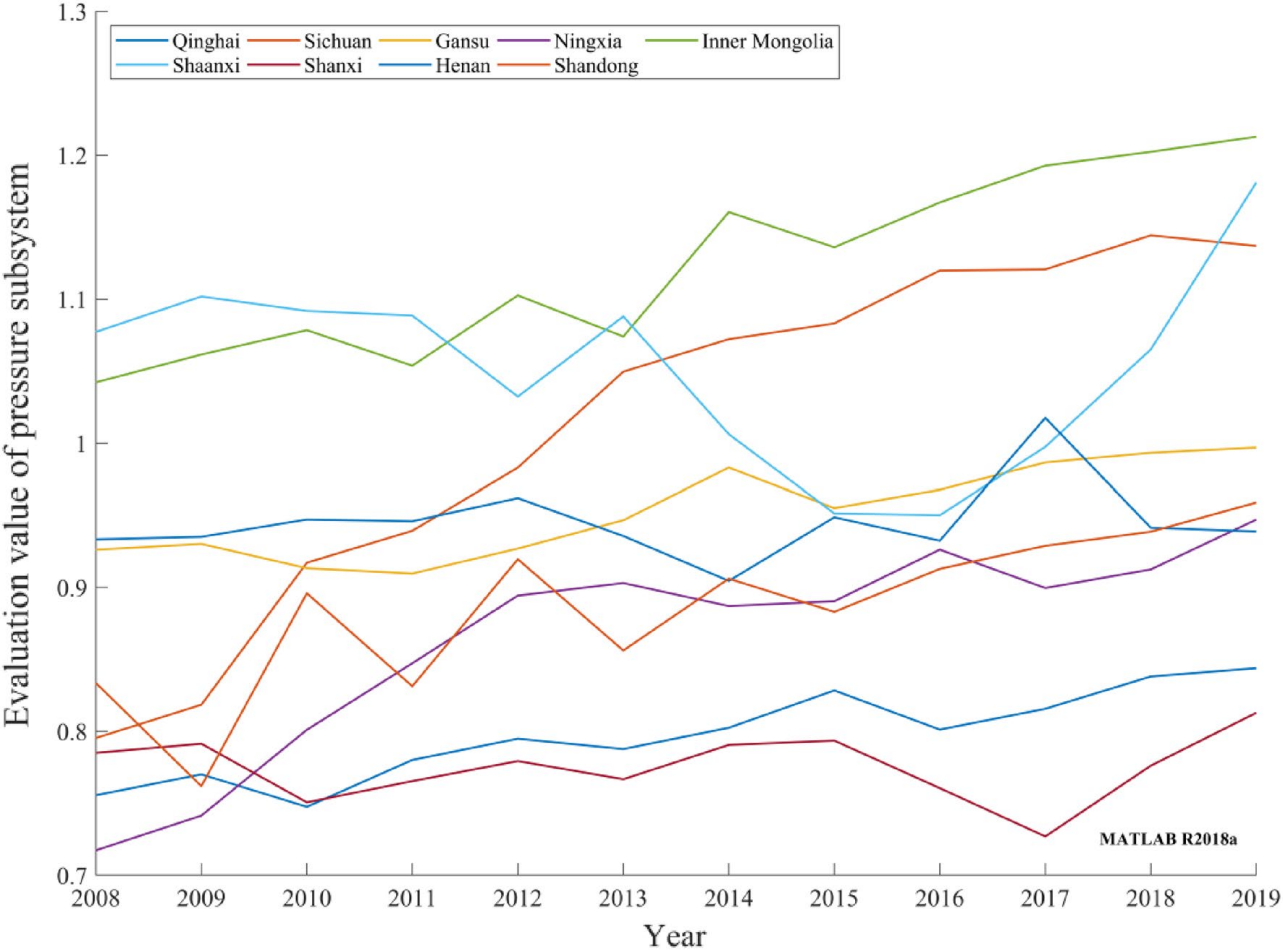
Evaluation values of provincial carbon-neutral pressure subsystems in the Yellow River basin during 2008–2019.
3. State subsystem
  Figure 5 exhibits the state subsystem evolution trend of the provincial carbon-neutral capacity in the Yellow River basin during 2008–2019. Under the dual action of driving and pressure, the evaluation value of the carbon-neutral state subsystem in Henan, Shandong, and Shanxi was at a low level. In the face of the environmental problems brought about by provincial economic development in the Yellow River basin, the effectiveness of the measures adopted is not obvious, thereby resulting in pressure on environmental resources. Existing governance measures cannot effectively improve the state of resources and the environment. The rationality of an industrial structure determines its level of economic quality. Industrial transformation and upgrading are conducive to improving the quality of the economy. When compared with the tertiary industry, the secondary industry in its growth stage has the characteristics of a high cost of unit economic growth. Inner Mongolia, Qinghai, and Gansu have relatively small proportions of secondary industry and large proportions of renewable energy in power generation. Therefore, these provinces maintain a good carbon-neutral state. Recently, Sichuan, Ningxia, and Shaanxi have actively developed emerging industries, strengthened ecological and environmental protection in the Yellow River basin, and formulated and implemented low-carbon development plans. These measures have effectively eliminated some of the negative impacts of development activities. Presently, the carbon neutrality states in most provinces in the Yellow River basin are alarming, and resource and environmental problems are evident. To improve their carbon-neutral states, the resources and environment of the Yellow River basin must be further and rationally utilized, treated, and protected. In general, China's carbon-neutral state subsystem in the Yellow River basin must be further improved.

**Figure 5:**
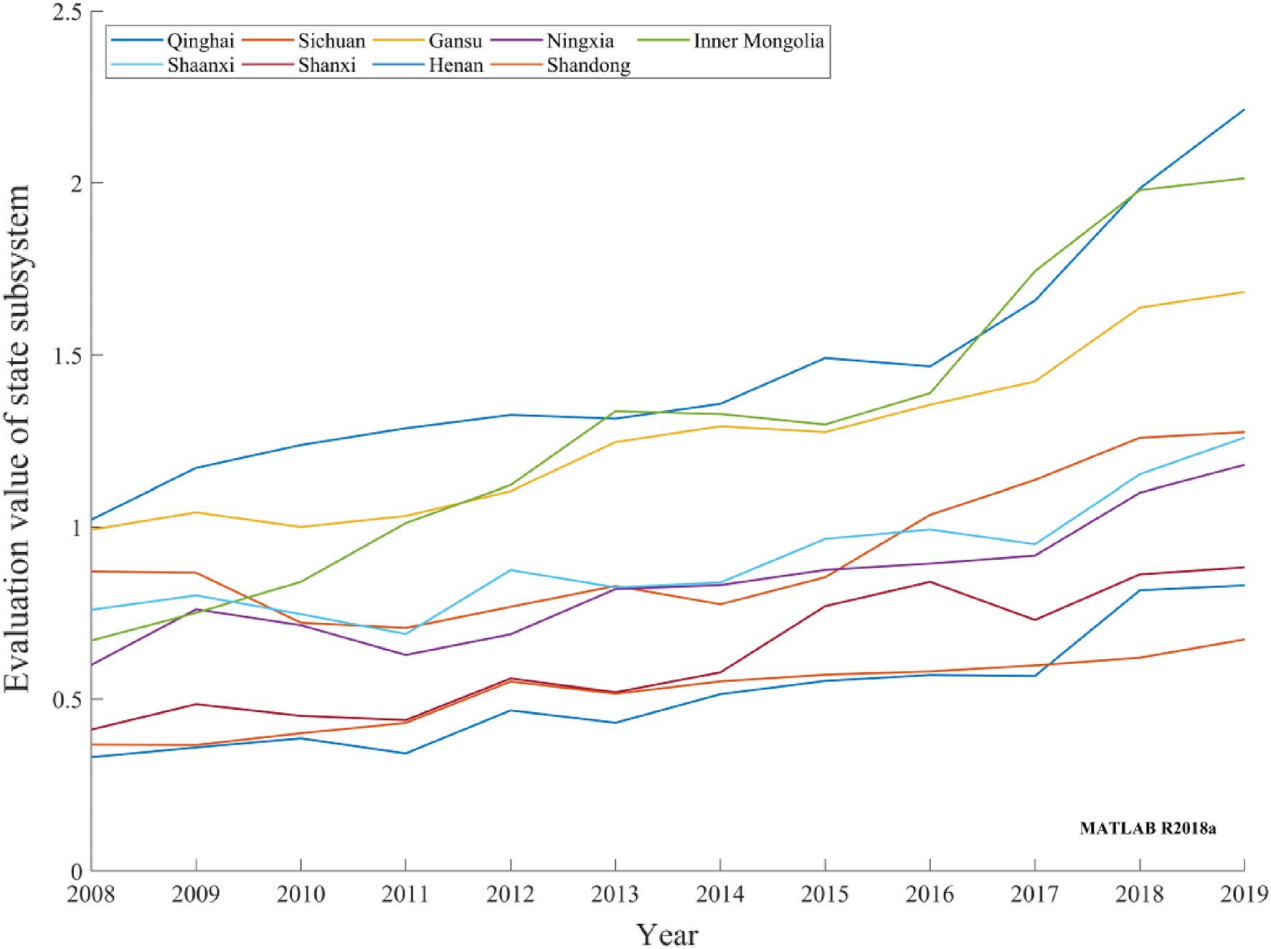
Evaluation values of provincial carbon-neutral state subsystems in the Yellow River basin during 2008–2019.
4. Impact subsystem
  Figure 6 depicts the impact subsystem evolution trends of the provincial carbon-neutral capacities in the Yellow River basin from 2008 to 2019. From the perspective of the environmental and resource impacts of provinces in the Yellow River basin, certain fluctuations are evident in the evaluation values of the carbon-neutral impact subsystems of provinces. Although the rates of increase in the temperature in all provinces are negative, the temperatures in all provinces in 2019 exhibited a significant increase compared with those in 2008. The Yellow River basin suffers from serious soil and water losses. Therefore, it has negatively impacted the trend of the carbon-neutral impact subsystem evaluation values. However, the characteristics of low carbon dioxide emissions and a low air comprehensive pollution index in Qinghai and Inner Mongolia ensure that the carbon-neutral impact subsystem in this region remains at a high level. Shaanxi, Henan, Sichuan, Gansu, Shanxi, Ningxia, and Shandong were negatively affected by provincial economic development. Owing to the excessive development of resources and emissions and wastewater in the process of economic development in each region, the evaluation value of the impact subsystem has decreased to a certain extent, thereby hindering any improvement of the evaluation value of the impact subsystem. Therefore, to mitigate the negative impact of provincial economic development in the Yellow River basin, it is necessary to establish a reasonable resource and environment utilization mechanism and strictly control the emissions of industrial pollutants.

**Figure 6:**
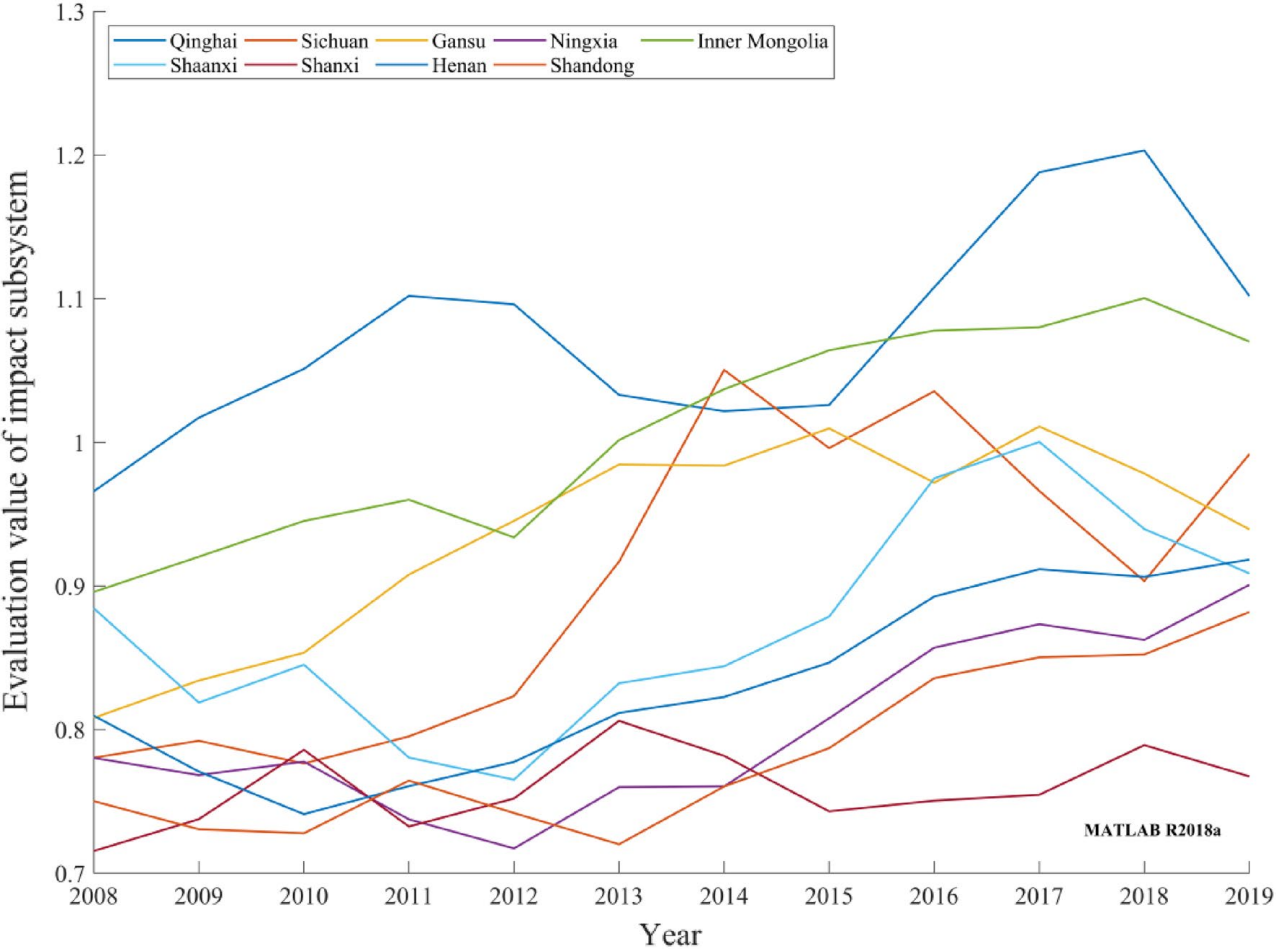
Evaluation values of provincial carbon-neutral impact subsystems in the Yellow River basin during 2008–2019.
5. Response subsystem
  Figure 7 depicts the response subsystem evolution trend of the provincial carbon-neutral capacity in the Yellow River basin from 2008 to 2019. Carbon-neutral driving indices and pressure indices varied across provinces, and the response degree of the measures adopted varied. However, the evaluation values of the response subsystem rapidly and steadily improved after wavy increases and decreases in the early stage. In Henan, Shaanxi, Shanxi, Shandong, and Sichuan, the government organized afforestation drives and invested efforts in scientific and technological research and development to improve the utilization rate of industrial waste and urban sewage and achieved good results. Ningxia and Gansu promoted public transport to implement a low-carbon economic development plan and promote the improvement of the evaluation value of the provincial carbon-neutral response subsystem in the Yellow River basin.

**Figure 7:**
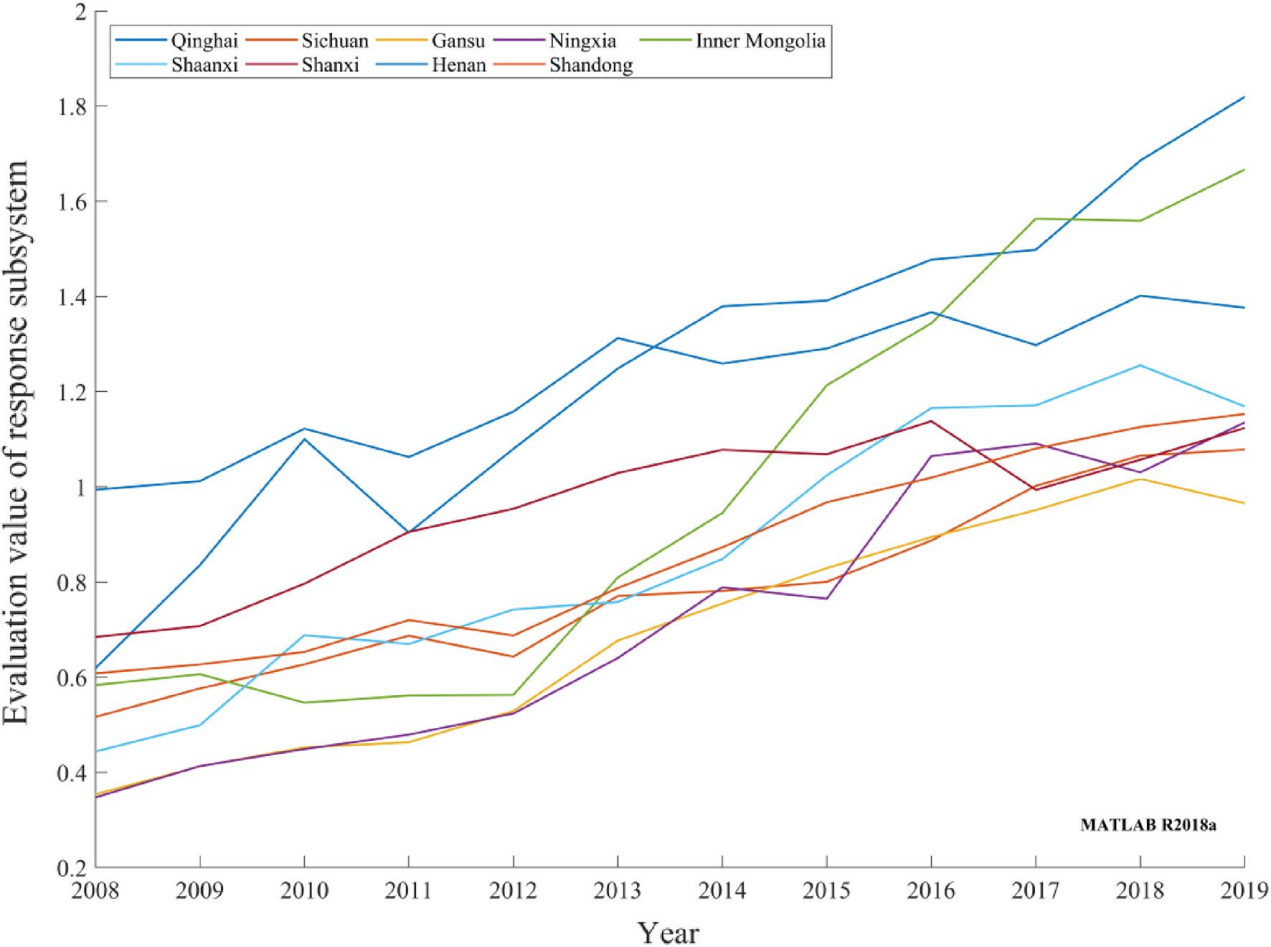
Evaluation values of provincial carbon-neutral response subsystems in the Yellow River basin during 2008–2019.

### Comprehensive evaluation and analysis of provincial carbon-neutral capacities in the Yellow River basin

**Figure 8:**
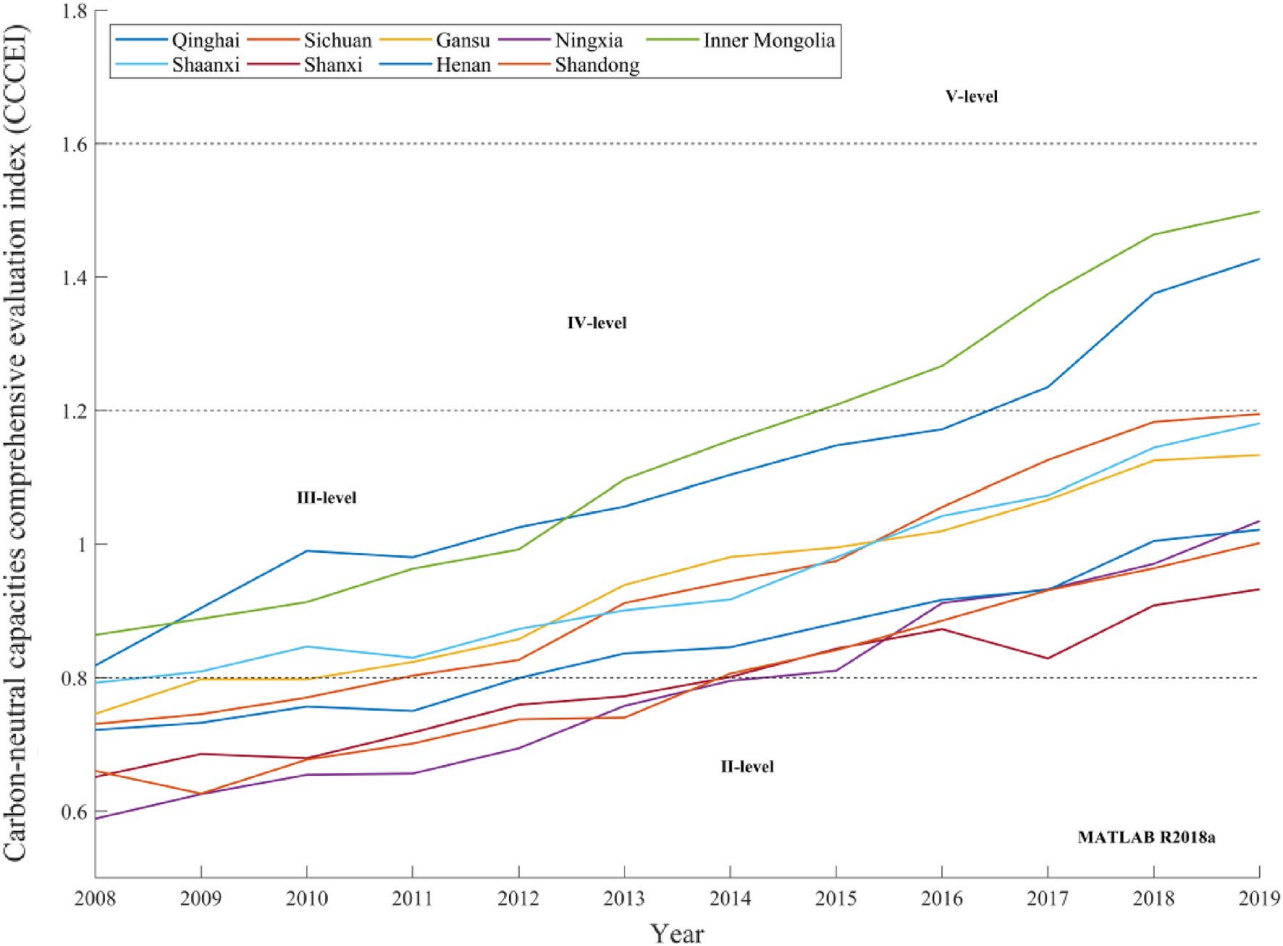
Provincial CCCEIs of the Yellow River basin from 2008 to 2019.

The provincial CCCEI in the Yellow River basin from 2008 to 2019 is depicted in Fig. 8. A radar map of the provincial CCCEI is presented in Fig. 9. The following features can be observed.

1. Overall, the provincial carbon-neutral capacities in the Yellow River basin were gradually enhanced.
  During 2008–2019, the provincial carbon-neutral capacities in the Yellow River basin were continuously enhanced, and the CCCEI exhibited an upward trend. Since 2013, the concepts of a low-carbon economy and sustainable development have attracted wide attention from society and governments at all levels. In 2013, *the State Council on Printing and Distributing the National Sustainable Development Plan for Resource-based Cities (2013–2020)* was issued. The development plan for sustainable economic development in the next seven years was introduced. From 2013 to 2019, nine provinces responded positively to state calls. Governments at all levels need to rationally develop and use environmental resources and strengthen environmental governance and protection in accordance with relevant work arrangements. The strong development of the response subsystem and gradual comprehensive and stable implementation of the regulation measures improved the quality of economic development, the pressure and negative impact on the ecological environment of the Yellow River basin decreased continuously, and the CCCEI increased rapidly.

**Figure 9:**
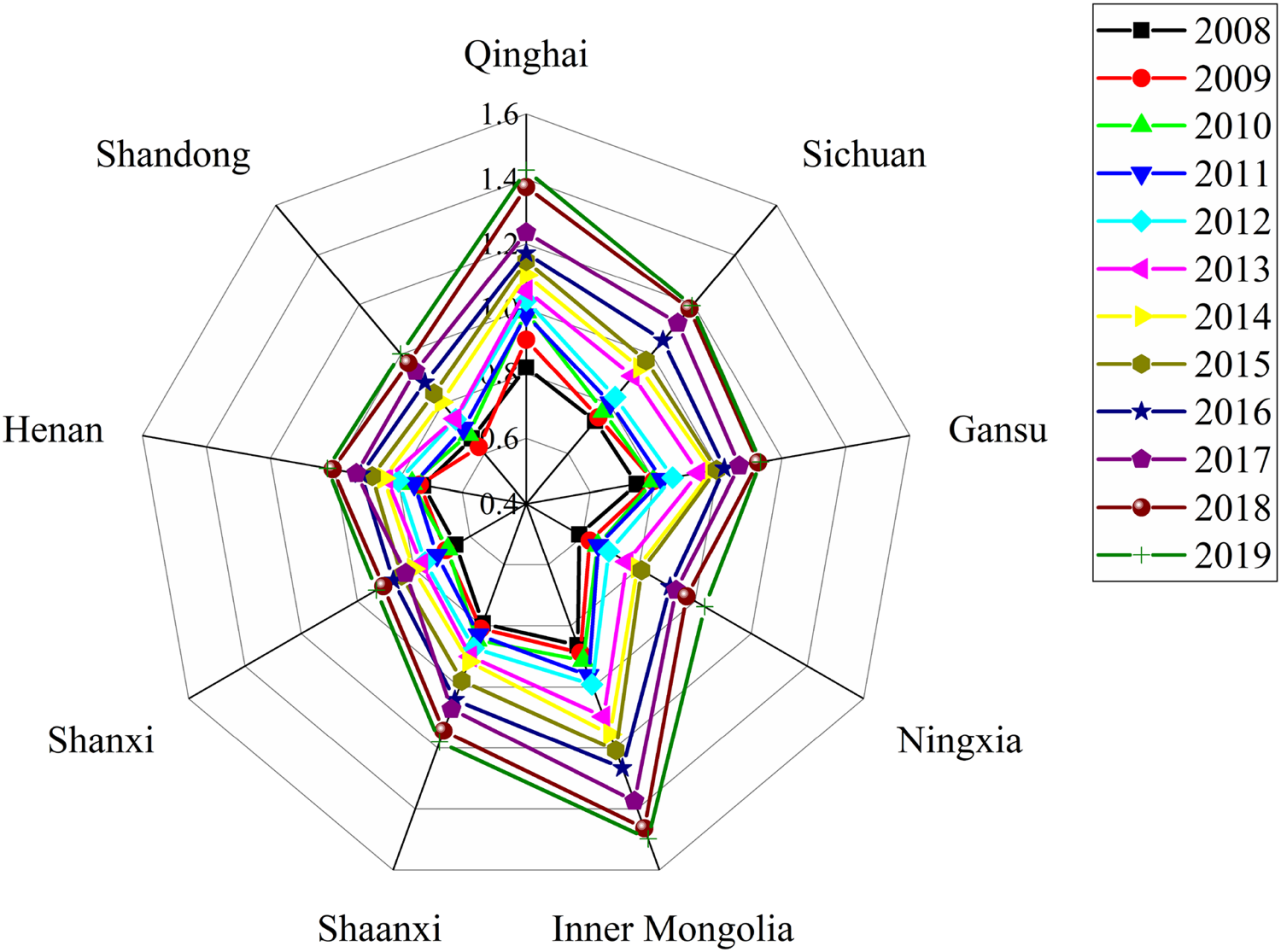
Radar map of provincial CCCEIs in the Yellow River basin from 2008 to 2019.
  However, driven by the ever-increasing population and economic aggregate, the increases in the coal-dominated industrial energy consumption structure and cars per capita ensure that the evaluation values of the pressure and impact subsystems fluctuate, thereby impeding the development of the provincial CCCEI in the Yellow River basin.
2. The development of carbon-neutral capacity in the Yellow River basin varies.
  Qinghai and Inner Mongolia have relatively good ecological environments, large vegetation coverage areas, and underdeveloped economies. Therefore, the provincial CCCEIs were above 0.8 in 2008.
  From 2008 to 2012, the provincial CCCEI values of Henan, Shanxi, Ningxia, and Shandong were less than 0.8. The overall levels of the carbon-neutral capacities of these provinces were level II, which were poor. Economic development leads to the destruction of resources and the environment. The evaluation values of the driving, pressure, and state subsystems were low.
  The provincial CCCEIs of Qinghai and Inner Mongolia were the first to attain level IV in 2017, which indicated that their carbon-neutral capacities were good. In 2019, the provincial CCCEIs of Sichuan, Shaanxi, and Gansu were above 1.1, i.e., slightly lower than the lower limit of the standard of IV. These provinces entered the initial stage of development with good carbon-neutral capacities. However, owing to the environmental damage and reduced green areas caused by economic development, the carbon-neutral capacities of Shanxi, Ningxia, Henan, and Shandong are relatively low. In 2019, their provincial CCCEIs were higher than 0.95 but lower than 1.1, thereby indicating their middle development stage of standard III.
3. The provinces in the Yellow River basin have significant development potential.
  During 2008–2019, the carbon-neutral capacities of provinces in the Yellow River basin achieved a grade leap. However, there is a huge scope for development beyond the upper limit of standard IV. Furthermore, no province has reached standard I, thereby leaving scope for further development of provinces. Ningxia and Shanxi are important coal carbon bases in China, and the coal energy industry is an important industry. With the development of green energy technology, the carbon-neutral capacities of these provinces will be significantly improved. Shandong and Henan Provinces have large populations. A small cultivated land area per capita, a small proportion of renewable energy power, and a large proportion of secondary industry lead to increased pressure on carbon-neutral capacities in these provinces. Therefore, it is necessary to increase the utilization of new energy, vigorously develop tertiary industry, strengthen carbon-capture technology, and strive to achieve the sustainable development goal of carbon neutrality at first.

### Discussion

1. A carbon-neutral-capacity-evaluation index system for the Yellow River basin is established based on the DPSIR model. The index system has three levels and 37 indicators^4,5,18,20–23,26^. The index system is scientific, complete, and easy to obtain and provides a basic framework for a comprehensive analysis of the causal relationship between carbon neutrality and social and economic activities in the Yellow River basin.
2. The global entropy method was used to calculate the capability evaluation value of each subsystem of the DPSIR, the CCCEI model of the Yellow River basin was constructed, and a classification standard for the carbon-neutral capability was proposed. This method can be used to perform quantitative analysis on each subsystem of the carbon-neutral capacity of each province in the Yellow River basin. It can dynamically describe the evolution trend of the subsystems and objectively measure the level and future development scope of the carbon-neutral capacity of each province. Compared with reference^7,13^, the practicability of DPSIR model and the scientificity of the result are further verified.
3. The comprehensive evaluation of carbon neutrality capacity in the whole Yellow River basin shows a significant upward trend, showing the spatial difference of the comprehensive evaluation of carbon neutrality capacity in the middle and lower reaches of the river basin than that in the upper reaches of the river basin. The evolution trend of spatial differences among each subsystem is basically consistent with the evolution trend of comprehensive evaluation.

## Conclusions and policy recommendations

### Conclusions

1. Based on DPSIR model and global entropy method, the comprehensive evaluation Index (CCCEI) of provincial carbon neutrality capacity in the Yellow River Basin was established to evaluate the carbon neutrality capacity of the Yellow River Basin from 2008 to 2019. The logical relationship among subsystems in DPSIR model and the spatial and temporal differences of each province are revealed, and the dynamic evolution trend of carbon neutral capacity of each province is analyzed.
2. From 2008 to 2019, the carbon neutrality capacity of the provinces in the Yellow River Basin was in a state of rapid development and achieved multi-level leapfrog, but there is still a great room for improvement. The carbon neutrality capacity of each subsystem shows two time differences: fluctuation and rise from 2008 to 2019. Driven by the continuous growth of population and economic aggregate, the continuous fluctuation of the evaluation index of pressure subsystem and influence subsystem hinders the consubstantial growth of the comprehensive evaluation index of carbon neutrality capacity of provinces in the Yellow River Basin to a certain extent.
3. The index system based on the DPSIR model is capable of evaluating the impacts of economic and social developments in various provinces on their carbon-neutral capacities, as well as those of positive measures adopted to achieve carbon neutrality. The proposed index system is broad based and can be extended to the carbon-neutral assessments of other river basins.

### Policy recommendations

Combined with the research and analysis, it is not difficult to see that the carbon neutrality capacity level of the provinces in the Yellow River Basin has been significantly improved. The evaluation of carbon neutrality capacity shows obvious regional heterogeneity and distinct regional characteristics. Accordingly, according to the provincial characteristics and differences carried by different river basins, policy suggestions are put forward to promote the carbon neutrality capacity of the Yellow River Basin as follows:

1. Provinces have actively developed emerging industries, strengthened ecological and environmental protection in the Yellow River basin, formulated and implemented low-carbon development plans, and effectively eliminated obstructive indicators. The carbon neutrality status of most provinces in the Yellow River Basin is relatively severe, and the resource and environment problems in the middle reaches are prominent. To improve the carbon neutrality capacity of the Yellow River basin, the resources and environment in the Yellow River basin need to be further rationally utilized, managed and protected. It is necessary to establish a reasonable resource and environmental utilization mechanism, strictly control the emission of industrial pollutants, advocate public transportation, promote the implementation of low-carbon economic development plans, and improve the comprehensive capacity of carbon neutrality in the provinces along the Yellow River Basin.
2. The distribution of the middle and lower reaches of the Yellow River provincial carbon neutral pressure generally larger, strengthen the response and the implementation of the policy, increasing factors of flow across the land, digging in the middle and lower reaches of the Yellow River and Yangtze river along the Banks of the leading type carbon neutral city experience, give full play to their ability of carbon neutral spillover effect and is a progressive effect, formation of the carbon cycle in the Yellow River urban agglomeration, It will drive the improvement of provincial carbon neutrality capacity in the form of urban agglomeration. Based on the development advantages of each region, the differences in factors hindering carbon neutrality shown by each region should be solved.
3. While dredging economic development, the government should constantly coordinate the contradiction between environmental protection and economic development, improve the overall coordinated development level at the material level such as industrial support and the accumulation of development factors, and formulate relevant policies according to local conditions.

## Data Availability

The data that support the findings of this study are available from the corresponding author upon reasonable request.
